# Mechanical, Physiological, and Perceptual Demands of Repeated Power Ability Lower-Body and Upper-Body Tests in Youth Athletes: Somatic Maturation as a Factor on the Performance

**DOI:** 10.3389/fpsyg.2020.01888

**Published:** 2020-07-31

**Authors:** Jorge Arede, Nuno Leite, Ben Bradley, Marc Madruga-Parera, Eduardo Saéz de Villarreal, Oliver Gonzalo-Skok

**Affiliations:** ^1^Research Center in Sports Sciences, Health Sciences and Human Development, CIDESD, University of Trás-os-Montes and Alto Douro, Vila Real, Portugal; ^2^A.F.C. Bournemouth, Bournemouth, United Kingdom; ^3^University School of Health and Sport (EUSES), University of Girona, Girona, Spain; ^4^Department of Football and Science, Pablo de Olavide University, Seville, Spain; ^5^Faculty of Health Sciences, University of San Jorge, Zaragoza, Spain

**Keywords:** technology, performance analysis, maximal power output, strength, high-intensity efforts

## Abstract

This study aims (a) to assess and compare the acute mechanical, physiological, and perceptual demands induced by a lower and upper body repeated power ability (RPA) protocols, and (b) to examine how the somatic maturation could predict training response in RPA. Thirteen young male basketball players (chronological age = 15.2 ± 1.1 years; height = 173.8 ± 9.5 cm; body mass = 71.7 ± 18.3 kg) were selected to perform the parallel Back Squat (BS), and Bench Press (BP) RPA protocols (3 blocks of 5 sets of 5 repetitions with 30 s and 3 min of passive recovery between sets and blocks, respectively). Mean propulsive power (MPP), accelerometer-based data, cardio-respiratory data, blood lactate, rate of perceived exertion (RPE) and muscle soreness were recorded. Somatic maturation was estimated according to the Khamis and Roche method. On the BS protocol, the mean oxygen uptake (VO_2_), heart rate (HR), and RPE were 1006.33 ± 481.85 ml/min., 133.8 ± 12.5 bpm, and 6.14 ± 0.98 A.U., while on the BP protocol, were 684.6 ± 246.3 ml/min., 96.1 ± 10.4 bpm, and 5.08 ± 1.44 A.U., respectively. Significant between-blocks differences were found for MPP, RPE, and blood lactate for both exercises. The BS implies higher cardio-respiratory and perceptual demands, though lower power production fluctuation and movement variability than the BP. The somatic maturation was a strong predictor of RPA-derived variables in BS. The MPP during all protocol, and the MPP during the Best Set were significant predictable by somatic maturation in both exercises. Mechanical, physiological and perceptual training demands are exercise and maturation dependent.

## Introduction

During team-sports matches, players are required to perform repeated bouts of high-intensity actions (HIA), such as sprinting, jumping, accelerations, decelerations, turns, and cutting interspersed with periods of low-to-moderate intensity actions (e.g., standing and walking) (Stojanović et al., 2018). Substantial decrements in HIA, mainly at the latter stages of matches (e.g., second half), have been typically reported in basketball (Stojanović et al., 2018) and other team-sports (Póvoas et al., 2012). As such, it is suggested that the ability to maintain HIA for the entire duration of a match is an important physical-fitness component in team-sports (Gabbett and Mulvey, 2008) particularly in basketball (Castagna et al., 2007). Thus, any strength and power training program with basketball players should aim to enhance the performance of HIA, and execute them efficiently throughout a match (Schelling and Torres-Ronda, 2016). Several training strategies have been recommended to optimize athletes’ ability to perform lower-body and upper-body actions in team-sports athletes (Gonzalo-Skok et al., 2016, 2017; Arede et al., 2019b). In this regard, the repeated-power ability (RPA) training consists of several blocks of sets of lower or upper-body maximal power with incomplete recovery periods between sets, that can concurrently target a wide variety of adaptations (e.g., cardio-respiratory, mechanical, neuromuscular) (Gonzalo-Skok et al., 2014, 2016, 2018). Besides being considered particularly effective to improve sprinting (1.6%), cutting (2.1%), and jumping (7.2–7.5%) in team-sport athletes (Gonzalo-Skok et al., 2016), this method has been recently proposed as an effective training modality to improve upper-body fatigue resistance (Gonzalo-Skok et al., 2018). The RPA training is largely explored in young and/or moderately resistance-trained basketball players during in-season (Gonzalo-Skok et al., 2016, 2018), nevertheless, additional evidence are required to clarify its perceptual and physiological demands to help practitioners to properly design training plans that include both in-court training sessions and the RPA training.

The effects of the RPA training are typically monitored and assessed through a linear transducer, and the variables often used include mechanical variables, such as the average power (Gonzalo-Skok et al., 2014, 2018), power fluctuations and the percentage of decrement in average power (Gonzalo-Skok et al., 2018). However, a previous study revealed most accurate information for strength and power assessment (Sánchez-Medina et al., 2010). The load that maximized the mechanical power output in bench press exercise was similar between mean propulsive power (MPP) (36.5% 1RM), and peak power (37.5% 1RM), but also lower comparing to the mean power (54.2%). Nevertheless, analyzing the load-power relationships according to three different measures (mean, peak, and MPP), the MPP was the most stable variable (*R*^2^ = 0.95), comparing to the mean power (*R*^2^ = 0.91), and peak power (*R*^2^ = 0.82) through the range of loads (10–100% 1RM) (Sánchez-Medina et al., 2010). Consequently, the mean propulsive power provided a better indicative of an individual’s true neuromuscular potential, comparing to mean and peak power (Sánchez-Medina et al., 2010). However, more studies are needed to confirm these findings. Previous studies showed that power production gradually decreases across the RPA protocol (Gonzalo-Skok et al., 2014), which suggest that the athletes experienced fatigue toward the end of the training sessions, which influenced muscle performance and, therefore, the ability to generate maximal power (Cormie et al., 2011a). Alterations at muscle properties and hormonal level due to fatigability mechanisms (Cormie et al., 2011a), could affect technical execution, however, this issue has been scarcely explored within strength and conditioning field. Recently, multiscale entropy measures, including the sample entropy (SampEn) have been explored to analyze the variability in accelerometer-based data derived of resistance training tasks (Moras et al., 2018). According to the findings, increments in movement variability resulted in higher values of SampEn (Moras et al., 2018), extending the understanding of the athlete’s movement during resistance training tasks. However, as far as we know, there is no study analyzing the movement variability through SampEn on upper-body nor lower-body repeated HIA.

A better understanding on how growth and maturational development interact with different training stimulus is essential for effective programming and, consequently, to improve athletic performance, throughout childhood and adolescence (Lloyd and Oliver, 2014). Adolescence is typically characterized by significant structural and functional modifications in the body, including the cardio-vascular response, expressed as the peak oxygen uptake, lactate production, carbon dioxide output, and minute ventilation (Armstrong et al., 2015). One of the best ways to know how these modifications occur requires the estimation of somatic maturation (Arede et al., 2019a). Previous studies analyzed how somatic maturation determined using both predicted age of peak height velocity, and percentage of predicted adult height (% PAH), affects strength-related parameters (Meylan C.M. et al., 2014; Meylan C.M.P. et al., 2014). Authors concluded as somatic maturation progresses athletes demonstrated higher values of maximum strength, maximal power output, and maximal force in concentric leg-press squat (Meylan C.M. et al., 2014; Meylan C.M.P. et al., 2014). Thus, somatic maturation helps practitioners to establish a better understanding of maturational status and therefore is beneficial to (i) design the appropriate training strategies, (ii) to optimize training adaptations and, consequently, and (iii) to achieve higher performance (Lloyd and Oliver, 2014; Haff and Triplett, 2016). However, scarce information is known about how somatic maturation is related with cardio-respiratory, mechanical, and neuromuscular parameters among both upper and lower-body repeated high-intensity protocols.

The aims of this study were (a) to assess and compare the mechanical, physiological, and perceptual demands induced by a lower and upper-body RPA, and (b) to examine how the somatic maturation could predict training response in RPA. We hypothesized that (i) training response differs according to the exercise, and (ii) somatic maturation predicts exercise response in RPA.

## Materials and Methods

### Procedures

All data were gathered in a single group using a descriptive design. It was taken by the same investigator to ensure testing accuracy and reliability. Data collection took place in the second month of the competitive season (November) after a 4-weeks pre-season period. Data collection included 6 consecutive sessions: first, subjects were recorded in anthropometrical data and assessed individual maximal heart rate (HR_max_) performing the Yo-Yo intermittent recovery test – Level 1 (Berkelmans et al., 2018). Second, an incremental load test to determine the load that maximized power output (Loadopt) in both BS and BP exercises was performed (two independent sessions). Then, one familiarization session with testing procedures (RPA protocol) was carried out. Finally, subjects undertook the RPA protocol (two independent sessions). The subjects had 48–72 h of rest between each session.

### Subjects

Twenty-two young male (U-14 to U-17) basketball players from a basketball academy were selected to participate in this study. Due to lack of interest (*n* = 6) or incomplete full protocol (*n* = 3), thirteen (chronological age = 15.2 ± 1.1 years; height = 173.8 ± 9.5 cm; body mass = 71.7 ± 18.3 kg; percentage of predicted adult height = 96.9 ± 3.9%) of the initial subjects completed the protocol. All participants had a minimum experience of 1 year in strength and power training, including parallel Back Squat (BS) and Bench Press (BP) exercises (training experience = 1.5 ± 0.5 years, range = 1–2 years). All players participated on an average of 6 h of basketball training, 2 strength/power sessions, and 1–2 competitive matches (regional level) per week. Each subject signed the approved assent form, while one parent or legal guardian also signed the approved consent form before the beginning of this investigation. The ethics committee of University of Trás-os-Montes and Alto Douro approved the present study, and it conformed to the recommendations of the Declaration of Helsinki (2013).

### Measures

#### Somatic Maturation

Height was recorded using a commercial portable stadiometer (Tanita BF-522W, Japan, nearest 0.1 cm). Body mass was estimated using the body fat monitor (Tanita BF-522W, Japan, nearest 0.1 kg). All measurements were taken following the guidelines outlined by the International Society for the Advancement of Kinanthropometry (ISAK) by the same researcher, who holds an ISAK Level 1 accreditation. Players’ height, body mass, chronological age and mid-parent height were used to predict the adult height of each player (Khamis and Roche, 1994). The height of the biological parents of each player were self-reported and adjusted for over-estimation using the previously established equations (Epstein et al., 1995). The current height of each player is then expressed as a percentage of adult height (% PAH), which can then be used as an index of somatic maturation (Roche et al., 2013). In these last years, the prediction of adult height using Khamis and Roche method is the most commonly used indicator of somatic maturation in sports field (Cumming et al., 2017; Malina et al., 2019). Moreover, this method is the most accurate to estimate age at peak height velocity, comparing to estimation based on generic age from longitudinal measures, and on the maturity offset equation (Parr et al., 2020). Individual maximal heart rate (HRmax). The HR_max_ was estimated trough the Yo-Yo intermittent recovery test – Level 1, considering the highest number of beats per minute recorded during the test. The test consists of consecutive series of 20-m shuttle runs at progressive velocities controlled by an audio beep interspersed by regular short rest periods of 10-s (Berkelmans et al., 2018). The test was finished when a subject failed to keep up with the audio beep in reaching the finish line on two separate or consecutive occasions.

#### Incremental Load Test

Incremental free parallel BS and BP load tests were used to determine the load that maximized mean propulsive power output (Loadopt) (Sánchez-Medina et al., 2010). The parallel BS was performed with plantar flexion to finish the movement, but jumping was not allowed. Subjects were instructed to squat down until the top of the thighs were parallel to the ground in a controlled manner and to perform the concentric phase of each repetition as fast as possible. The BP started with the bar at arm’s length and lowered the bar until the chest was touched lightly without bounding the bar. Subjects were instructed to perform the concentric phase of each repetition as fast as possible, without a pause between eccentric and concentric phases, and the eccentric phase in a controlled manner (i.e., approximately 2 s). Initial load was set at 20 kg for all subjects and was progressively increased in 10 kg until the attained mean propulsive velocity (MPV) was lower than 0.99 m⋅s^–1^ in parallel BS, and lower than 0.50 m⋅s^–1^ in BP (Sánchez-Medina et al., 2010). Thereafter, load was adjusted with smaller increments (2.5–5 kg), individually for each subject. For the lighter loads (BS = MPV > 1.0 m⋅s^–1^; BP = MPV > 0.97 m⋅s^–1^) three attempts were executed at each load; two for the medium (BS = 0.57 m⋅s^–1^ ≤ MPV ≤ 0.99 m⋅s^–1^; BP = 0.50 m⋅s^–1^ ≤ MPV ≤ 0.97 m⋅s^–1^); and only one for the heaviest loads (BS = MPV < 0.57 m⋅s^–1^; BP = MPV < 0.50 m⋅s^–1^) (Sánchez-Medina et al., 2010). A 3-min rest was established for lighter and medium loads, and 5-min for the heaviest loads (Sánchez-Medina et al., 2010). Only the best repetition at each load, according to the criteria of the highest mean propulsive power output (MPP), was considered for analysis (Sánchez-Medina et al., 2010). The MPP showed an almost perfect individual load-power relationship (*R*^2^ = 0.95), which allowed to determine the power output at each percentage of one maximum repetition (% 1RM) for each subject were observed (Sánchez-Medina et al., 2010). The test was performed using barbell standard 20-kg (BOXPT Equipment, Póvoa de Varzim, Portugal) and the barbell velocity was recorded with a SmartCoach Power Encoder linear transducer (SmartCoach Europe AB, Stockholm, Sweden), and computed in SmartCoach software into a personal computer (ASUS, model A541U).

#### Repeated-Power-Ability (RPA) Training Protocol

The RPA protocol consisted of 3 blocks of 5 sets of 5 repetitions with 30-s of passive recovery between sets and 3-min of passive recovery between blocks in both free parallel BS and BP exercises using Loadopt. The concentric phase was executed as fast as possible, and the eccentric phase was performed at slower velocity (i.e., self-selected and never exceeding 3-s) than the concentric phase, neither bouncing on the chest nor jumping. The protocol was performed using barbell standard 20-kg (BOXPT Equipment, Póvoa do Varzim, Portugal) and the barbell velocity was recorded with a SmartCoach Power Encoder linear transducer (SmartCoach Europe AB, Stockholm, Sweden), and computed in the SmartCoach software into a personal computer (ASUS, model A541U). Percentage of MPP decrement was calculated using the following formula: % Dec = 100 – (MPP_mean_/MPP_best_) × 100). Intraset power fluctuation (FLUC), was considered and calculated as follows: FLUC = (SD of MPP in each set/mean of each set) × 100, for the best set, the last set, and all protocol (i.e., mean of 3 blocks).

#### Total Acceleration (AcelT)

The acceleration in the anteroposterior axis (Z), in the transverse or lateral axis (X), and vertical axis (Y) for the overall movement was measured using an inertial measurement unit (WIMU, Realtrack Systems, Almeria, Spain) attached to the barbell. The WIMU unit integrates accelerometer, gyroscope, and magnetometer with sampling frequency for 3-axis of 100 Hz (Muyor et al., 2018). The main variable used was the acceleration magnitude or resultant vector of acceleration which is called total acceleration (AcelT) (García-Rubio et al., 2018). AcelT was computed using the following formula: AcelT = $\sqrt{x^{2}+y^{2}+}z^{2}$. Coefficient of variation (CV), and Sample Entropy (SampEn) for AcelT (Moras et al., 2018) were computed using SPRO Software v1.0.0 (Realtrack Systems, Almeria, Spain). CV was calculated using the following formula: CV = $\frac{\sigma }{\mu }$. SampEn (m,r,n) is defined as the negative natural logarithm of the conditional probability that two sequences, similar form points (length of the vector to be compared), remain similar at the next point m + 1 (Silva et al., 2017). The values used to calculate SampEn were 2 to vector length (m) and 0.2 ± SD to the tolerance (r) (Silva et al., 2017). Values of SampEn range from zero toward infinity, where values close to zero were indicative of higher regularity in AcelT, while the higher the SampEn, the more unpredictable the AcelT (Silva et al., 2017).

#### Cardio-Respiratory Variables

Cardio-respiratory measures were collected continuously with breath-by-breath method using a reliable and valid automated open-circuit gas analysis system (K5, Cosmed Srl, Rome, Italy), during the RPA protocol (Perez-Suarez et al., 2018). Pulmonary ventilation (VE), oxygen uptake (VO_2_) and carbon dioxide production (VCO_2_) were assessed. Careful calibrations of flow sensors and gas analyzers were performed before each measurement according to the manufacturer’s instructions. Thus, immediately before each test session, gas analyzers were calibrated using ambient air (20.93% oxygen and 0.03% carbon dioxide) and with a standard gas mixture (containing 16.00% oxygen and 5.02% carbon dioxide). The turbine flow meter, a bidirectional turbine with an optoelectronic reader, was calibrated with a 3-L syringe (Cosmed Srl, Rome, Italy) and used for the determination of minute ventilation. The wearable equipment was positioned on the subjects and the bidirectional turbine was attached to a facemask (Hans Rudolph Inc., Shawnee, KS, United States) covering both the mouth and the nose.

#### Blood Lactate

Before and after the RPA testing protocol (1-, 3-, 5-, and 7-min.), capillary blood samples were obtained from the earlobe of participants and analyzed using a reliable and valid handheld lactate analyzer (Accutrend Plus; Boehringer; Mannheim, Germany) (Bonavolontà et al., 2009). The responsible researcher carefully cleaned, disinfected and dried the subjects’ ear lobe before the blood collection. Subjects’ skin was punctured with a lancet and the first drop of blood was placed straight on the strip. The highest value was considered for further analysis.

#### Perceptual Response (RPE)

After the completion of each block of RPA testing protocol, all participants were requested to quantify the RPE through the Borg CR10 scale (Borg and Kaijser, 2006).

#### Muscle Soreness

Muscle soreness was determined in quadriceps muscle during a body-weight parallel BS after the lower-body exercise, and in pectoralis major muscle (on the BP), when the muscle was palpated using the Visual Analogue Scale (0: no pain at all, 10: worst pain ever), immediately after the completion of the testing protocol.

### Statistical Analyses

Data are presented as mean ± SD. Normality of data distribution and sphericity were confirmed using the Shapiro-Wilk statistic and Levene’s Test for equality of variances. Repeated measures univariate analysis of variance (ANOVA) were conducted for differences in different variables between all blocks. Independent *t*-tests were used to compare dependent variables between the training conditions (BS vs. BP). Effect Sizes (ES) analysis was also computed using a published spreadsheet in Microsoft Excel (Hopkins, 2007). Threshold values for Cohen’s d for ES statistics were 0–0.2 trivial, >0.2–0.6 small, >0.6–1.2 moderate, >1.2–2.0 large, and >2.0 very large (Hopkins et al., 2009). A stepwise multiple regression was used to determine a predictive equation to estimate RPA-derived variables from % PAH alone. The level of statistical significance was set at *p* < 0.05. All statistical analyses were performed using SPSS software (version 24 for Windows; SPSS Inc., Chicago, IL, United States).

## Results

The results obtained in BS were 46.3 ± 12.2 l/min., 1006.3 ± 481.8 ml/min., 133.8 ± 12.5 bpm, and 3.36 ± 1.78 mmol/l for VE, VO_2_, HR and Lactate, respectively (Table 1). Also, the mean RPE and muscle soreness were 6.14 ± 0.98 A.U., and 6.83 ± 0.94 A.U., respectively (Table 1). Regarding the BP, the mean VE, VO_2_, HR, Lactate were 25.7 ± 7.2 l/min., 684.6 ± 246.3 ml/min., 96.11 ± 10.4 bpm, and 4.11 ± 1.43 mmol/l, respectively (Table 2). Also, the mean RPE and muscle soreness were 5.08 ± 1.44 A.U., and 6.25 ± 0.75 A.U., respectively (Table 2).

**TABLE 1:** Overall results from the Parallel Back-Squat RPA protocol.

Variables			
1RM (kg)	88.71 ± 18.16	VO_2_ (ml/min.)	1006.33 ± 481.85
% 1RM	65.53 ± 9.47	VO_2_ rel (ml/kg/min.)	13.78 ± 5.49
MPO (W)	467.83 ± 118.03	VCO_2_ (ml/min.)	603.77 ± 562.90
Loadopt (kg)	55.92 ± 13.73	VCO_2_ rel (ml/kg/min.)	8.13 ± 6.75
Best Set (W)	414.32 ± 100.26	HR (bpm)	133.77 ± 12.49
Last Set (W)	334.68 ± 72.96	HRmáx (%)	68.22 ± 7.91
MPP (W)	359.24 ± 84.76	Lactate (mmol/l)	3.36 ± 1.78
Dec (%)	13.16 ± 5.68	ACELT CV (a.u.)	0.22 ± 0.03
FLUCB (a.u.)	7.85 ± 2.53	ACELT SampEn (a.u.)	0.48 ± 0.14
FLUCL (a.u.)	8.35 ± 2.25	RPE (a.u.)	6.14 ± 0.98
FLUCM (a.u.)	7.89 ± 1.52	Muscle Soreness (a.u.)	6.83 ± 0.94
VE (l/min.)	46.29 ± 12.20		

*1RM, one maximum repetition; % 1RM, percentage of one maximum repetition; MPO, maximal power output; Loadopt, load which maximized propulsive power; MPP, mean propulsive power; Dec, decrement in mean propulsive power; FLUCB, power fluctuation in the best set; FLUCL, power fluctuation in the last set; FLUCM, mean power fluctuation; VE, minute ventilation; VO_2_, oxygen uptake; VCO_2_, carbon dioxide output; HR, heart rate; HRmáx, percentage of maximum heart rate; ACELT, total acceleration; CV, coefficient of variation; SampEn, sample entropy; RPE, rate of perceived exertion.*

**TABLE 2:** Overall results from the Bench-Press RPA protocol.

Variables			
1RM (kg)	57.05 ± 14.50	VO_2_ (ml/min.)	684.60 ± 246.32
% 1RM	44.80 ± 9.06	VO_2_ rel (ml/kg/min.)	9.25 ± 2.33
MPO (W)	312.24 ± 90.10	VCO_2_ (ml/min.)	525.39 ± 327.62
Loadopt (kg)	25.83 ± 9.00	VCO_2_ rel (ml/kg/min.)	6.86 ± 3.42
Best Set (W)	281.91 ± 76.20	HR (bpm)	96.11 ± 10.40
Last Set (W)	211.17 ± 65.87	HRmáx (%)	48.89 ± 5.45
MPP (W)	243.32 ± 69.40	Lactate (mmol/l)	4.11 ± 1.43
Dec (%)	13.75 ± 5.60	ACELT CV (a.u.)	0.40 ± 0.11
FLUCB (a.u.)	7.60 ± 3.46	ACELT SampEn (a.u.)	0.72 ± 0.26
FLUCL (a.u.)	10.40 ± 2.96	RPE (a.u.)	5.08 ± 1.44
FLUCM (a.u.)	9.81 ± 3.54	Muscle Soreness (a.u.)	6.25 ± 0.75
VE (l/min.)	25.75 ± 7.16		

*1RM, one maximum repetition; % 1RM, percentage of one maximum repetition; MPO, maximal power output; Loadopt, load which maximized propulsive power; MPP, mean propulsive power; Dec, decrement in mean propulsive power; FLUCB, power fluctuation in the best set; FLUCL, power fluctuation in the last set; FLUCM, mean power fluctuation; VE, minute ventilation; VO_2_, oxygen uptake; VCO_2_, carbon dioxide output; HR, heart rate; HRmáx, percentage of maximum heart rate; ACELT, total acceleration; CV, coefficient of variation; SampEn, sample entropy; RPE, rate of perceived exertion.*

Results of the inferential analysis between exercises is displayed in Table 3. FLUCL (*p* < 0.05), FLUCM (*p* < 0.05), ACELT CV (*p* < 0.0001), and ACELT SampEn (*p* < 0.05), were significantly higher in BP than BS. Furthermore, VE (*p* < 0.0001), VO_2_ (*p* < 0.05), VO_2_ rel (*p* < 0.05), HR (*p* < 0.0001), and RPE (*p* < 0.05) were significantly higher in BS than BP.

**TABLE 3:** Comparison between parallel Back Squat and Bench Press protocols.

Variables	*p*	η2	Differences	Variables	*p*	η2	Differences
Dec (%)	0.801	0.01 *No effect*		VCO_2_ (ml/min.)	0.661	0.02 *No effect*	
FLUCB (a.u.)	0.854	0.00 *No effect*		VCO_2_ rel (ml/kg/min.)	0.595	0.03 *No effect*	
FLUCL (a.u.)	0.037	0.34 *Moderate*	BP > BS	HR (bpm)	0.000	0.93 *Strong*	BS > BP
FLUCM (a.u.)	0.046	0.32 *Moderate*	BP > BS	Lactate (mmol/l)	0.315	0.09 *Minimum*	
VE (l/min.)	0.000	0.89 *Strong*	BS > BP	ACELT CV (a.u.)	0.000	0.79 *Strong*	BP > BS
VO_2_ (ml/min.)	0.024	0.38 *Moderate*	BS > BP	ACELT SampEn (a.u.)	0.032	0.35 *Moderate*	BP > BS
VO_2_ rel (ml/kg/min.)	0.021	0.40 *Moderate*	BS > BP	RPE (a.u.)	0.017	0.42 *Moderate*	BS > BP

*Dec, decrement in mean propulsive power; FLUCB, power fluctuation in the best set; FLUCL, power fluctuation in the last set; FLUCM, mean power fluctuation; VE, minute ventilation; VO_2_, oxygen uptake; VCO_2_, carbon dioxide output; HR, heart rate; ACELT, total acceleration; CV, coefficient of variation; SampEn, sample entropy; RPE, rate of perceived exertion; BS, parallel back squat; BP, parallel bench press.*

Significant between-block differences were found for MPP (*p* < 0.0001), ACELT CV (*p* < 0.05), RPE (*p* < 0.0001), and blood lactate (*p* < 0.05), on the BS (Table 4). Also, significant between-block differences were found for MPP (*p* < 0.01), RPE (*p* < 0.0001), and blood lactate (*p* < 0.0001), on the BP (Table 5).

**TABLE 4:** Between-blocks comparisons in the mechanical and perceptual variables – Parallel Back Squat.

Variable	Block 1	Block 2	Block 3	Repeated measures ANOVA		Effect Size (ES) 95% Confidence Interval		
				*p*	η2	Block 1 vs. Block 2	Block 1 vs. Block 3	Block 2 vs. Block 3
MPP (W)	384.87 ± 94.33	354.37 ± 85.96	338.48 ± 77.13	0.000*#$	0.64 *Moderate*	0.27 [0.13; 0.41] *Small*	0.44 [0.30; 0.57] *Small*	0.15 [0.05; 0.25] *Trivial*
FLUCM (a.u.)	7.57 ± 2.21	7.79 ± 1.56	8.31 ± 2.53	0.517	0.04 *No effect*	−0.22 [−0.84; 0.40] *Small*	−0.29 [−1.10; 0.52] *Small*	−0.13 [−0.76; 0.50] *Trivial*
ACELT CV (a.u.)	0.23 ± 0.04	0.22 ± 0.04	0.21 ± 0.04	0.032*#	0.45 *Moderate*	0.49 [0.11; 0.87] *Small*	0.71 [0.26; 1.16] *Moderate*	0.23 [−0.06; 0.51] *Small*
ACELT SampEn (a.u.)	0.46 ± 0.25	0.52 ± 0.18	0.45 ± 0.18	0.632	0.04 *No effect*	−0.38 [−1.07; 0.31] *Small*	0.02 [−0.82; 0.87] *Trivial*	0.36 [−0.23; 0.94] *Small*
RPE (a.u.)	4.83 ± 1.34	6.17 ± 0.94	7.42 ± 1.00	0.000*#$	0.82 *Strong*	−1.62 [−2.28; −0.96] *Very Large*	−3.11 [−3.96; −2.27] *Very Large*	−1.30 [−1.81; −0.78] *Very Large*

**Variable**	**Before**	**After**		***p***	**η2**	**Before vs. After**		

Lactate (mmol/l)	1.63 ± 0.63	3.36 ± 1.76		0.013	0.44 *Moderate*	−1.43 [−2.29; −0.58] *Very Large*		

* Significant difference (p < 0.05) between Block 1 and 2; # Significant difference (p < 0.05) between Block 1 and 3; $ Significant difference (p < 0.05) between Block 2 and 3; MPP, mean propulsive power; FLUCM, mean power fluctuation; ACELT, total acceleration; CV, coefficient of variation; ApEn, aproximate entropy; SampEn, sample entropy; RPE, rate of perceived exertion.

**TABLE 5:** Between-blocks comparisons in the mechanical and perceptual variables – Parallel Bench Press.

Variable	Block 1	Block 2	Block 3	Repeated measures ANOVA		Effect Size (ES) 95% Confidence Interval		
				*p*	η2	Block 1 vs. Block 2	Block 1 vs. Block 3	Block 2 vs. Block 3
MPP (W)	257.85 ± 75.19	244.52 ± 69.12	227.60 ± 67.42	0.001*$	0.49 *Moderate*	0.13 [0.03; 0.24] *Trivial*	0.33 [0.12; 0.53] *Small*	0.20 [0.09; 0.31] *Small*
FLUCM (a.u.)	9.25 ± 3.20	10.14 ± 4.61	10.05 ± 3.85	0.528	0.06 *Minimum*	−0.11 [−0.42; 0.20] *Trivial*	−0.18 [−0.59; 0.23] *Small*	−0.05 [−0.55; 0.45] *Trivial*
ACELT CV (a.u.)	0.40 ± 0.10	0.41 ± 0.12	0.40 ± 0.11	0.516	0.06 *Minimum*	−0.06 [−0.22; 0.10] *Trivial*	0.02 [−0.20; 0.25] *Trivial*	0.08 [−0.11; 0.27] *Trivial*
ACELT SampEn (a.u.)	0.73 ± 0.36	0.74 ± 0.35	0.69 ± 0.20	0.813	0.02 *No effect*	−0.05 [−0.48; 0.39] *Trivial*	0.00 [−0.91; 0.91] *Trivial*	0.07 [−0.81; 0.96] *Trivial*
RPE (a.u.)	3.83 ± 1.59	5.08 ± 1.56	6.33 ± 1.61	0.000*#$	0.72 *Strong*	−0.92 [−1.38; −0.46] *Moderate*	−1.65 [−2.36; −0.94] *Large*	−0.69 [−1.02; −0.37] *Moderate*

**Variable**	**Before**	**After**		***p***	**η2**	**Before vs. After**		

Lactate (mmol/l)	1.84 ± 0.80	4.11 ± 1.43		0.000	0.70 *Strong*	−2.38 [−3.25; −1.52] *Very Large*		

* Significant difference (p < 0.05) between Block 1 and 2; # Significant difference (p < 0.05) between Block 1 and 3; $ Significant difference (p < 0.05) between Block 2 and 3; MPP, mean propulsive power; FLUCM, mean power fluctuation; ACELT, total acceleration; CV, coefficient of variation; ApEn, aproximate entropy; SampEn, sample entropy; RPE, rate of perceived exertion.

**FIGURE 1:**
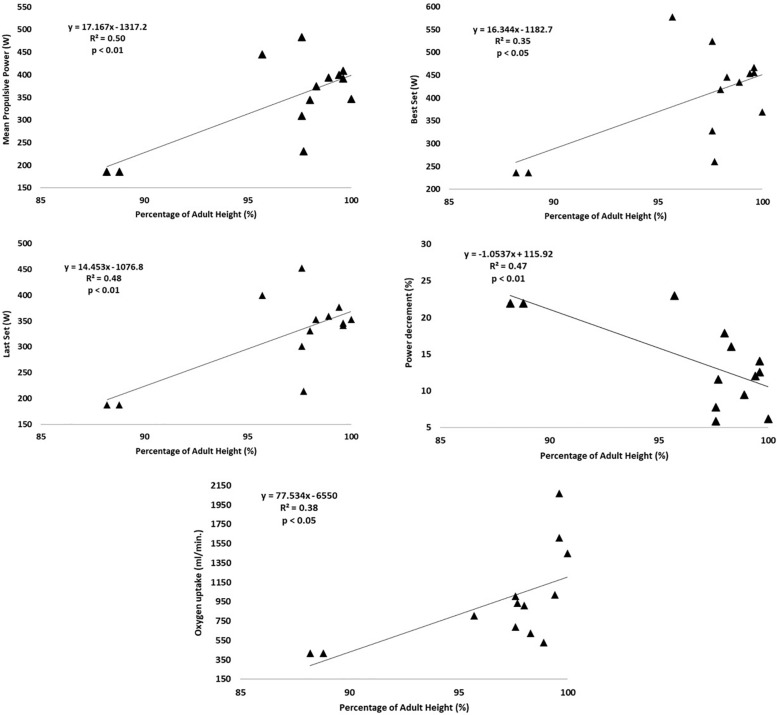
Linear relationship between **(A)** Mean Propulsive Power and% PAH, **(B)** Best Set and% PAH, **(C)** Last Set and% PAH, **(D)** Power decrement and% PAH, and **(E)** Oxygen uptake and% PAH.

**FIGURE 2:**
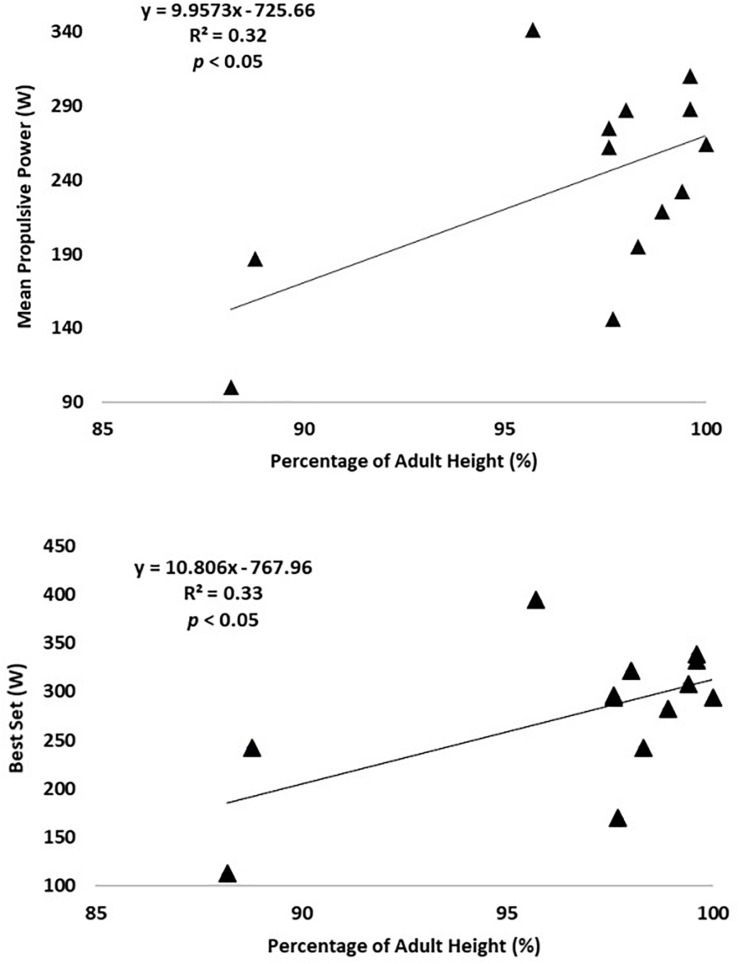
Linear relationship between **(A)** Mean Propulsive Power and %PAH, and **(B)** Best Set and %PAH.

Multiple regression analysis revealed% PAH as significant predictor of MPP (*p* < 0.05; *R*^2^ = 0.50), Best set (*p* < 0.05; *R*^2^ = 0.35), Last set (*p* < 0.01; *R*^2^ = 0.48), % Dec (*p* < 0.01; *R*^2^ = 0.47), and oxygen uptake (*p* < 0.05; *R*^2^ = 0.38), on the BS (Figure 1). Also, on the BP, % PAH as significant predictor of MPP (*p* < 0.05; *R*^2^ = 0.32), and Best set (*p* < 0.05; *R*^2^ = 0.33) (Figure 2).

## Discussion

The aims of this study were (a) to assess and compare the acute mechanical, physiological, and perceptual demands induced by a lower and upper-body repeated power ability (RPA) bouts, and (b) to examine how the somatic maturation could predict training response in RPA. We found that lower-body RPA training protocol requires higher cardio-respiratory and perceptual demands, and lower power production fluctuation and movement variability than the upper-body training strategy. It was also observed that some lower- and upper body RPA-derived variables were predicted using somatic maturation, i.e., the present findings support our hypothesis that training response differs according to the exercise, and somatic maturation predicts exercise response in RPA, particularly in lower-body RPA.

Maximal muscular power is associated with enhanced athletic performance and the improvement of this capacity requires the use of complex movements performed with loads ranging from 50 to 90% of 1RM, for lower-body exercises, and with loads ranging from 30 to 45% of 1RM, for upper-body exercises (Cormie et al., 2011b). The RPA includes repeated bouts of short high-intensity efforts with sub-maximal loads (45–66% of 1RM). The short and maximal sustained efforts (i.e., 5–10 s at 90–100% of maximum power output) are associated with a lower contribution of aerobic mechanisms (4%) and a higher contribution of anaerobic mechanisms (96%) (Haff and Triplett, 2016). The total duration of the whole RPA protocol (≈15 min.) suggests that the oxidative system also contributes to the energy production, with light stress of cardiorespiratory (48.89–68.2%HR_máx_) and glycolytic systems (3.31–4.11 mmol/l) (Haff and Triplett, 2016). Previous studies in basketball-match showed that heart rate ranged from 66.7 to 89.1% of HR_máx_, and the blood lactate concentration from 2.7 to 6.8 mmol/l (Stojanović et al., 2018).

The onset of blood lactate accumulation (OBLA) which represents the concentration of blood lactate at 4 mmol/L is a well-established indicator of exercise intensity (Bompa and Haff, 2009; Haff and Triplett, 2016). Training at intensities near or above the OBLA promotes that the lactate accumulation occurs later at a higher exercise intensity, due to reduced catecholamine release at high exercise intensities, and increased mitochondrial content (Haff and Triplett, 2016). Because of this, occurs greater production of adenosine triphosphate (ATP) through aerobic mechanisms, and greater%VO_2max_ with lower blood lactate accumulation (Haff and Triplett, 2016). It seems that RPA shows some physiological correspondence with basketball-match demands, i.e., periods of high-intensity activity are interspersed with periods of low- to moderate-intensity activities. Performing RPA efforts, athletes could experience similar blood lactate accumulation to a basketball-match in a shorter period of exposure (≈15 min. vs. 40 min.), generating metabolic adaptations which may be determinant for on-court performance.

The present results indicate that lower-body RPA training protocol implies higher cardio-respiratory (VE, VO2, VO2 rel, and HR) and perceptual demands (RPE), and also lower power production fluctuation (FLUCL and FLUCM) and movement variability (ACELT CV and SampEn) than the upper-body training strategy. The physiological demands are directly related to the amount of muscle tissue activated by the exercise (Haff and Triplett, 2016). The parallel BS involving a larger muscle mass or a greater work level than BP is likely to be associated with a higher VE, VO2, VO2 rel, and HR (Haff and Triplett, 2016). Maximal muscular power is accompanied by fast-twitch muscle fibers involvement (Cormie et al., 2011a). However, these type of muscle fibers are particularly fatigable, because they contain a low content of myoglobin, relatively few mitochondria, and relatively few blood capillaries (Cormie et al., 2011a). In addition, the rest interval affects the restoration of ATP and phosphocreatine, the clearance of fatigue-inducing substrates, and the restoration of force production capacity (Bompa and Haff, 2009). Thus, to completely restore the force- and power-generating capacity, the between-sets recovery should be within 2 to 5 min. (Bompa and Haff, 2009). In the present protocol, subjects rested 30 s between sets, and 3 min between each block of 5 sets, affecting power-generating capacity, particularly in that exercise which involves a smaller amount of muscle mass (i.e., BP) as there is not enough between-blocks recovery to be fully recovered. In fact, altered muscle properties (i.e., release and pump back of calcium in skeletal muscle) could negatively affect maximal muscular power (Cormie et al., 2011a) and, consequently, increase power fluctuations (i.e., FLUCL and FLUCM) or movement variability (i.e., ACELT CV and SampEn). The present study failed to demonstrate any differences in blood lactate values between RPA protocols. In RPA greater emphasis is placed on mechanical stress due to the use of a lower number of repetitions per set (i.e., lower time under tension) with a short inter-set rest interval (Zatsiorsky and Kraemer, 2006). In contrast, resistance exercise protocols which include higher number of repetitions per set (i.e., higher time under tension) generate higher metabolic stress, resulting of the accumulation of metabolites, particularly lactate (Schoenfeld, 2013). In this regard, previous study revealed that higher values of blood lactate and significant between-exercises differences (BS and BP) occurred in resistance exercise protocols including higher number of repetitions per set (i.e., 10–12) at higher intensity (i.e., close of maximum predicted number of repetitions per set) (Sánchez-Medina and González-Badillo, 2011). However, in resistance exercise protocols including lower number of repetitions per set at lower intensity, lower values of blood lactate and no significant between exercises were reported (Sánchez-Medina and González-Badillo, 2011). Thus, we can claim that RPA involves mainly stress of the phosphagen system, and decreased carbohydrate metabolism, based on decreased accumulation of blood lactate, irrespective of resistance exercise. This hypothesis seems to be reinforced by the fact that adolescents have reduced capacity to utilize carbohydrate during submaximal exercise compared to young adult counterparts (Stephens et al., 2006). In fact, less mature subjects have lower number of key enzymes in the glycolytic pathways, attenuated sympathetic activity, and lower muscle glycogen content which result in lesser capacity to utilize carbohydrate, and consequently lower accumulation of blood lactate (Stephens et al., 2006). In both exercises, the MPP decreased across the RPA, with significant differences between blocks 1, 2, and 3. These results are in accordance with previous studies using a RPA training protocol (Gonzalo-Skok et al., 2014). During the first set, subjects performed higher concentric average power, while average power gradually decreases in the subsequent sets (412.5 > 391.7 > 378.8 > 367 > 358.2 W) (Gonzalo-Skok et al., 2014). The RPA training protocol consists of maximal power training with incomplete recovery periods (Gonzalo-Skok et al., 2016). The rest interval affect the restoration of adenosine triphosphate and phosphocreatine, the clearance of fatigue-inducing substrates, and the restoration of force production capacity (Bompa and Haff, 2009). However, short rest intervals coupled with high volumes of training generate physiological adaptations, such as capillary density, mitochondrial density, and buffer capacity which results in an endurance performance increased (Bompa and Haff, 2009). In fact, the enhanced ability to tolerate repeated sprints after RPA training might be associated to these kind of adaptations (Gonzalo-Skok et al., 2016). Considering the MPP decrements, we can claim that the present training protocol reproduces in controlled setting the substantial decrements in HIA at the latter stages of a match.

The multiple regression analysis confirmed somatic maturation (expressed as% PAH) as a significant predictor of MPP and Best set for both exercises and for Last set, % Dec, and oxygen uptake only in the BS exercise. Thus, more advanced somatic maturation corresponds to higher values of the above-mentioned variables, with the exception of% Dec. Growth and maturation are characterized by significant changes in muscle structure, size, and metabolism, but also in neuromuscular system (Van Praagh and Doré, 2002; Lloyd and Oliver, 2014; Armstrong et al., 2015; Cumming et al., 2017). More matured subjects have a concomitant higher muscle fiber size, type II fiber distribution, and also higher levels of testosterone secretion (Van Praagh and Doré, 2002). Increased testosterone induces selective hypertrophy of type II fibers which could confer an advantage in power activities (Van Praagh and Doré, 2002; Cormie et al., 2011a), expressing by higher MPP and Best Set, as occurred in the present study.

Furthermore, a combination of changes in anatomical, metabolic and hematological factors underpin the ability of cardiopulmonary system to cope with increased exercise intensities, in later stages of maturation (Lloyd and Oliver, 2014). In fact, increased lungs size, heart size and number of alveoli results in higher values of VO_2_ during exercise (Lloyd and Oliver, 2014). The present results support the hypothesis that somatic maturation could promote different responses to the RPA training, due to metabolic, muscular and neuromuscular factors.

Training methods which can concurrently stimulate different capacities are most efficient to catalyze improvements in HIA, in more mature athletes (Lloyd and Oliver, 2014; Gonzalo-Skok et al., 2016, 2018). In this regard, a previous study with U-18 elite basketball players (Gonzalo-Skok et al., 2016), showed considerable improvements in repeated sprint ability (ES = 0.49) and repeated change of direction (ES = 0.60) after a low-volume and frequency lower-limbs RPA training. In the same line, another study made with U-17 elite basketball players (Gonzalo-Skok et al., 2018) showed meaningful improvements in BP performance at 1RM (ES = 0.26) and light to submaximal loads (20–80% 1RM, ES = 0.37–0.89). Improved lower and -upper body HIA performance might offer important competitive advantage to youth basketball players. Thus, RPA training protocol should be a suitable strategy to improve both upper- and lower-body HIA in youth athletes, particularly among more matured athletes. Limitations for interpreting the data of this study include the relatively small sample size (*n* = 13). Despite the small sample size, gold-standard technology was used to analyze key variables, biological maturation was assessed (in previous studies it was not taken into account (Gonzalo-Skok et al., 2014, 2016, 2018), and significant inferences were observed. However, given the current lack of knowledge regarding the physiological, and perceptual demands of RPA, this this study provides support for future investigations to clarify RPA training effects in the setting of the team-sports. Additional research with larger sample, players of different sport, age, gender, and playing standards should be conducted before the applicability of the current results can be generalized. Future studies should be conducted to analyze the short- and medium-term effects in lower-body (jumping, sprinting, and cutting) and upper-body (throwing and shooting) HIA following the RPA protocol. Finally, it would be interesting to analyze other performance variables, such as surface electromyography or biochemical markers (e.g., creatine kinase, and testosterone: cortisol).

## Conclusion

The RPA focuses on maximal repeated power training with incomplete recovery periods in between sets, using multiple joint exercises, provides a high neuromuscular, and lower metabolic and perceptual stimulus. It has practical applicability due to the existence of many sports that include strength and power expressions under predominantly aerobic requirements (e.g., soccer, basketball). So, training together both qualities would optimize athletic performance similarly to traditional training in a more time-efficiently manner. Considering the present findings, it would be expected that the RPA is a time effective way of generating positive neuromuscular and physiological adaptations in youth athletes. However, somatic maturation explains more aspects of exercise response during lower body RPA protocol than during upper body RPA protocol. Higher levels of testosterone secretion during adolescence, promotes the growth of contractile tissue and the skeletal development, and consequently increased strength (Round et al., 1999; Van Praagh and Doré, 2002). These effect of testosterone is particularly higher on the quadriceps muscle (Round et al., 1999), determinants on the execution of parallel BS. Whereas somatic maturation can be a confound variable in the optimization of training adaptations, practitioners should assess technical competence, but also somatic maturation, before designing and prescribe this type of training protocols for young athletes. During upper body RPA protocol occurred higher power fluctuation and movement variability may demonstrate the importance of mastering technical competency, comparing to the lower body protocol. Previous studies demonstrated that power fluctuation is independent of overall power capacity (Gonzalo-Skok et al., 2014), and muscle activation during bench press exercise at different intensities is distinct according to the experience, particularly of main stabilizer muscle (i.e., medial deltoid) (Shick et al., 2010). If practitioners intend train simultaneously distinct qualities through upper body RPA protocol should mainly consider the technical competency. Moreover, upper body RPA protocol may be especially useful to optimize athletic performance, when players are going through developmental phases in which they may be more susceptible to injury in lower limbs (i.e., the pubertal growth spurt).

## Data Availability Statement

The raw data supporting the conclusions of this article will be made available by the authors, without undue reservation.

## Ethics Statement

The studies involving human participants were reviewed and approved by the University of Trás-os-Montes and Alto Douro Research Ethics Committee. Written informed consent to participate in this study was provided by the participants, and if necessary, the participants’ legal guardian/next of kin.

## Author Contributions

JA, NL, and OG-S: conceptualization, investigation, and methodology. JA: data curation, formal analysis, software, and writing – original draft. JA, NL, BB, MM-P, ES, and OG-S: writing – review and editing. All authors contributed to the article and approved the submitted version.

## Conflict of Interest

The authors declare that the research was conducted in the absence of any commercial or financial relationships that could be construed as a potential conflict of interest.
